# Sevoflurane Pre-conditioning Ameliorates Diabetic Myocardial Ischemia/Reperfusion Injury Via Differential Regulation of p38 and ERK

**DOI:** 10.1038/s41598-019-56897-8

**Published:** 2020-01-08

**Authors:** Dina Xie, Jianli Zhao, Rui Guo, Liyuan Jiao, Yanqing Zhang, Wayne Bond Lau, Bernard Lopez, Theodore Christopher, Erhe Gao, Jimin Cao, Xinliang Ma, Yajing Wang

**Affiliations:** 1 Department of Emergency Medicine, Thomas Jefferson University, Philadelphia, PA USA; 2 Department of Cardiovascular Surgery, Tianjin Medical University General Hospital, Tianjin, China; 3 Department of Physiology, Shanxi Medical University, Shanxi, China; 4 Center for Translational Research, Temple University, Philadelphia, PA USA

**Keywords:** Interventional cardiology, Myocardial infarction

## Abstract

Diabetes mellitus (DM) significantly increases myocardial ischemia/reperfusion (MI/R) injury. During DM, cardioprotection induced by conventional pre-conditioning (PreCon) is decreased due to impaired AMP-activated protein kinase (AMPK) signaling. The current study investigated whether PreCon with inhaled anesthetic sevoflurane (SF-PreCon) remains cardioprotective during DM, and identified the involved mechanisms. Normal diet (ND) and high-fat diet (HFD)-induced DM mice were randomized into control and SF-PreCon (3 cycles of 15-minute period exposures to 2% sevoflurane) groups before MI/R. SF-PreCon markedly reduced MI/R injury in DM mice, as evidenced by improved cardiac function (increased LVEF and ±Dp/dt), decreased infarct size, and decreased apoptosis. To determine the relevant role of AMPK, the effect of SF-PreCon was determined in cardiac-specific AMPKα2 dominant negative expressing mice (AMPK-DN). SF-PreCon decreased MI/R injury in AMPK-DN mice. To explore the molecular mechanisms responsible for SF-PreCon mediated cardioprotection in DM mice, cell survival molecules were screened. Interestingly, in ND mice, SF-PreCon significantly reduced MI/R-induced activation of p38, a pro-death MAPK, without altering ERK and JNK. In DM and AMPK-DN mice, the inhibitory effect of SF-PreCon upon p38 activation was significantly blunted. However, SF-PreCon significantly increased phosphorylation of ERK1/2, a pro-survival MAPK in DM and AMPK-DN mice. We demonstrate that SF-PreCon protects the heart via AMPK-dependent inhibition of pro-death MAPK in ND mice. However, SF-PreCon exerts cardioprotective action via AMPK-independent activation of a pro-survival MAPK member in DM mice. SF-PreCon may be beneficial compared to conventional PreCon in diabetes or clinical scenarios in which AMPK signaling is impaired.

## Introduction

Diabetic patients endure increased mortality following acute myocardial infarction^1^. Conventional preconditioning (short-term ischemic episodes before an extended ischemic period) has been extensively studied in hearts achieved from animals and patients. Although conventional preconditioning significantly rescues damaged heart tissue, its clinical application remains a significant challenge^2^. Volatile anesthetics (such as sevoflurane) are myocardial protective^3–5^, and are widely used in the induction of patients experiencing coronary artery bypass grafting (CABG) surgery in the operative and perioperative period. However, clinical trials have noted conflicting results in patients with obesity and diabetes^6^. Determining the etiology of the discrepancy between clinical and experimental data may reveal an important mechanistic understanding of the value of preconditioning by volatile anesthetics, and may yet yield their clinical applicability in diabetic patient cardioprotection.

Both basic and clinical studies demonstrate the susceptibility of the diabetic heart to MI/R injury due to impaired AMP-activated protein kinase (AMPK, a key regulator of metabolism) signaling^7,8^. A recent scientific report demonstrated sevoflurane is an AMPK activator^9^. Whether any potential benefit of sevoflurane preconditioning against MI/R injury in a diabetic heart is associated with AMPK remains unknown.

The present study determined whether sevoflurane preconditioning (SF-PreCon) in a high-fat diet induced diabetic model diminishes MI/R-induced cardiac injury. Employing AMPKα2 dominant negative expressing (AMPK-DN) mice, we determined the influence of AMPK signaling on the observed effects.

## Results

### Sevoflurane preconditioning improved cardiac function and reduced infarct size in high-fat diet induced diabetic (DM) mice post MI/R

**Figure 1:**
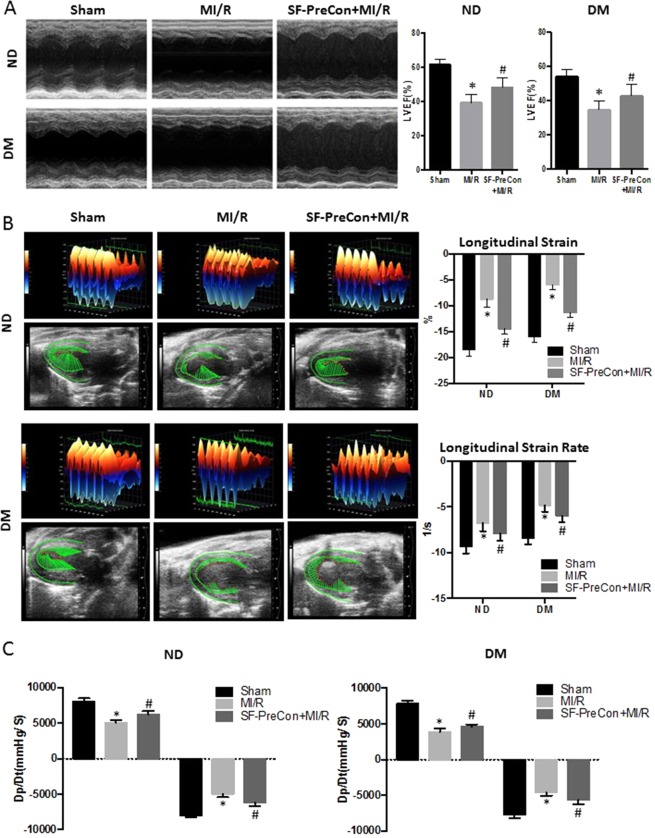
SF-PreCon increased cardiac function in ND and HFD DM mice after MI/R. (**A**) Sevoflurane preconditioning improved cardiac function in ND and HFD DM mice, evidenced by echocardiography. (**B**) Three-dimensional regional wall velocity diagrams showing contraction (orange/positive values) or relaxation (blue/negative values) of 3 consecutive cardiac cycles. Vector diagrams showing the direction and magnitude of endocardial contraction at midsystole. Global averages of strain and strain rate measured in the longitudinal axes across the LV endocardium. (**C**) ±Dp/dt (via hemodynamics assay) of Sham, MI/R, SF-PreCon+MI/R groups. Abbreviations: ND, Normal diet; HFD, High fat diet; DM, diabetes.

Normal diet (ND) or high-fat diet (HFD)-induced DM mice were randomized to control and SF-PreCon groups prior to MI/R. SF-PreCon significantly improved cardiac function in ND mice, as evidenced by increased left ventricular ejection fraction (LVEF, +8.9% compared to MI/R, P < 0.05 Fig. 1A) and increased ±Dp/dt (23.7% and 23.4% compared to MI/R, P < 0.05, Fig. 1C). Strain analysis was performed on long-axis B-mode images to determine whether regions injected with Pre-SFCon exhibited improved contractile activity. Representative 3-dimensional wall velocity diagrams for 3 consecutive cardiac cycles are shown from animals at baseline (Sham, MI/R, and SF-PreCon treatment groups, Fig. 1B). All hearts from all groups exhibit uniform and synchronous contraction and relaxation at baseline across the LV endocardium. In the MI/R group, there was marked reduction in wall velocity across the endocardium of the infarct-related anterior wall. Pre-SFCon treated animals exhibited markedly increased wall velocity and strain (Fig. 1B top).

**Figure 2:**
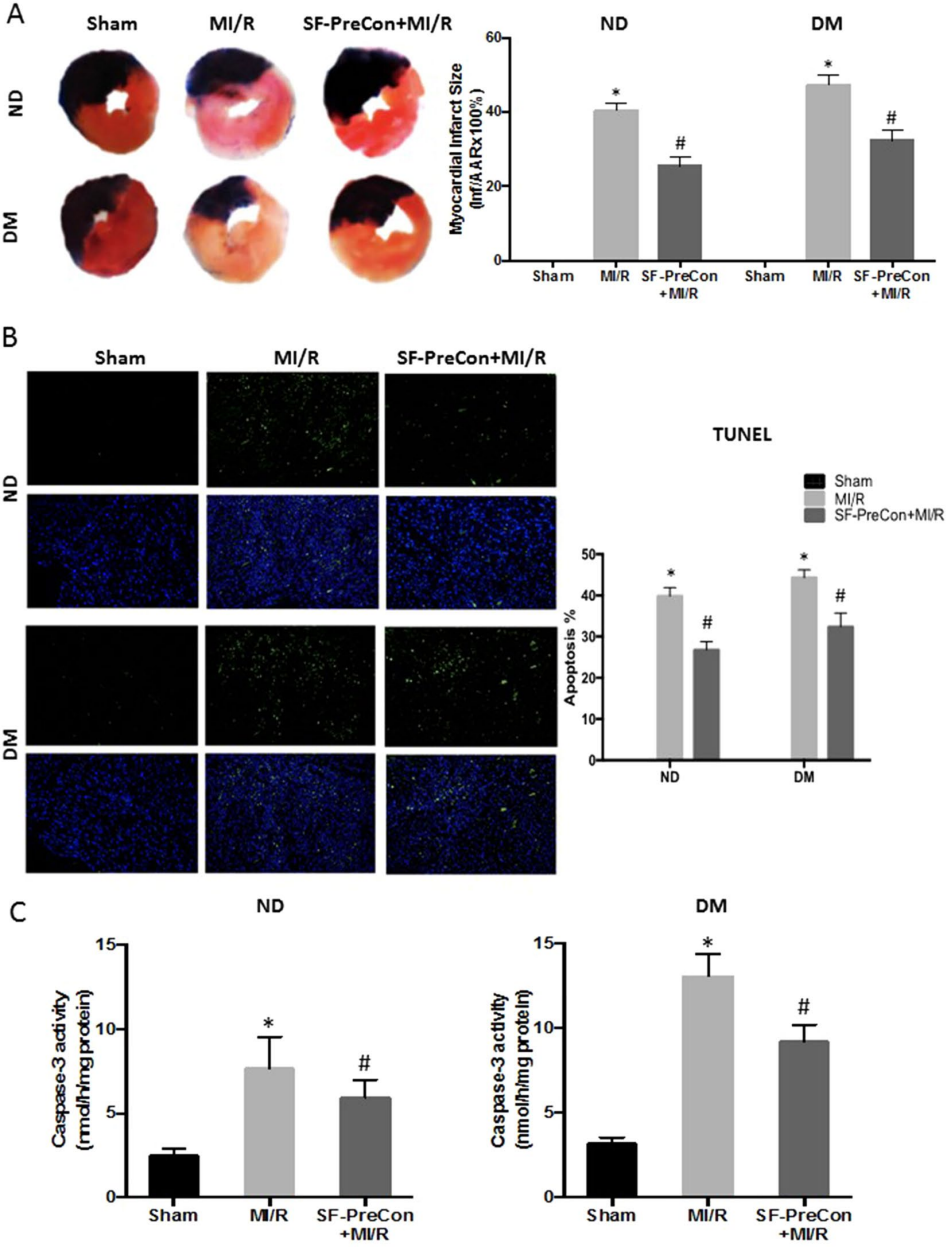
SF-PreCon reduced MI/R injury in ND and HFD DM mice. (**A**) Representative images of cardiac sections of (left to right) Sham, MI/R, SF-PreCon+MI/R groups. Infarct size was diminished in SF-PreCon group compared to MI/R group, both in ND and HFD DM mice after MI/R injury. (**B**) TUNEL staining (**C**) Caspase-3 activity assay (n = 6–10, *p < 0.05 compared with Sham, #p < 0.05 compared to MI/R).

SF-PreCon markedly decreased both infarct size (−15.1% compared to MI/R, P < 0.05, Fig. 2A) and apoptotic cell death detected by terminal deoxynucleotidyl transferase dUTP nick-end labeling (TUNEL) asssay (−13% TUNEL stain positive cells compared to MI/R, P < 0.05, Fig. 2B and −22.7% caspase-3 activity compared to MI/R, P < 0.05, Fig. 2C). We next determined whether SF-PreCon mediated cardioprotection remains present in DM mice subjected to MI/R. SF-PreCon significantly augmented cardiac function (LVEF: +8.2% compared to MI/R in DM, P < 0.05, Fig. 1A; ±Dp/dt: 22.1% and 21.8% increase compared to MI/R in DM, P < 0.05, Fig. 1C;), decreased infarct size (−14.8% compared to MI/R in DM, P < 0.05, Fig. 2A), and decreased apoptosis (−11.9% TUNEL stain positive cells compared to MI/R, P < 0.05, Fig. 2B; −29.6% caspase-3 activity compared to MI/R in DM, P < 0.05, Fig. 2C). Meanwhile, the cardiac function was obviously augmented in both Longitudinal strain and strain rate (Fig. 1B down). Together, these results support SF-PreCon decreases MI/R-induced cardiac dysfunction in both ND and DM mice.

### SF-PreCon-mediated cardioprotection intact in AMPK-DN mice

**Figure 3:**
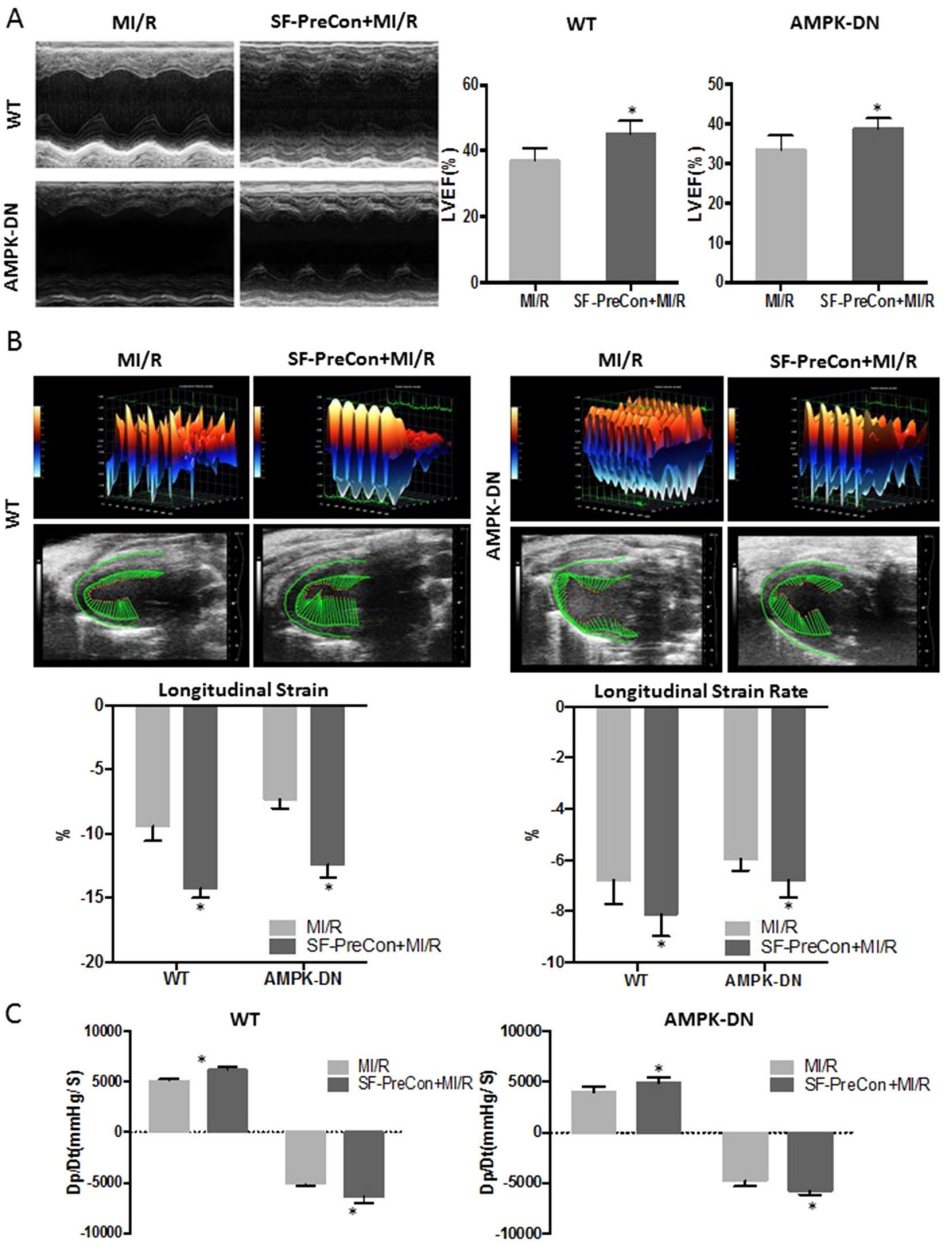
SF-PreCon increased cardiac function in ND and AMPK-DN mice. To determine the role of AMPK in cardioprotection by SF-PreCon, a cardiac specific AMPKα2 dominant negative mouse (AMPK-DN) was employed. (**A**) SF-PreCon significantly increased heart function both in WT and AMPK-ND mice after MI/R, evidenced by echocardiography. (**B**) Three-dimensional regional wall velocity diagrams and vector diagrams. (**C**) Hemodynamic measurements. Abbreviations: WT, Wild type.

**Figure 4:**
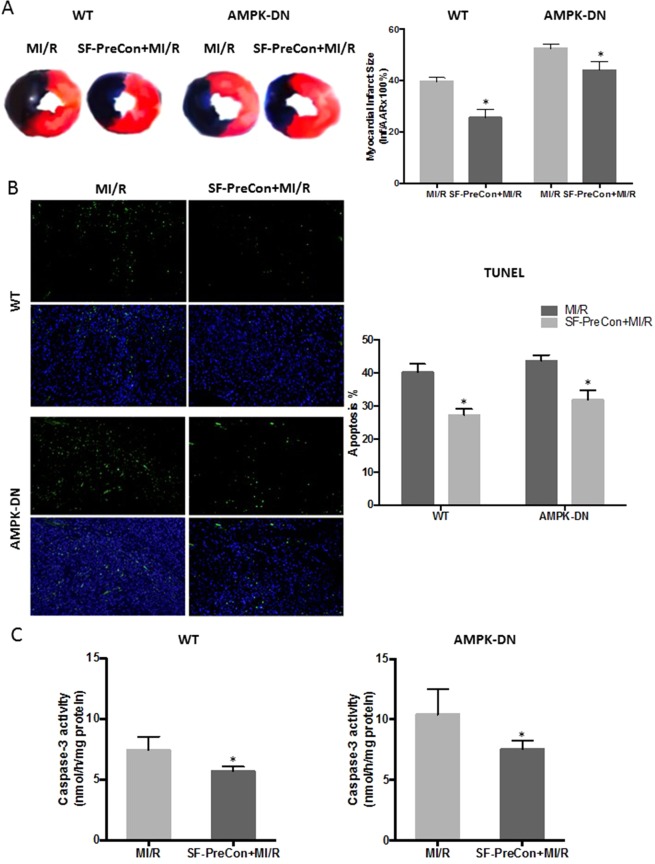
SF-PreCon reduced MI/R injury in ND and AMPK-DN mice. SF-PreCon significantly reduced MI/R injury both in WT and AMPK-DN mice. Showing (**A**) Infarct size. (**B**) TUNEL staining. (**C**) Caspase-3 activity. (n = 10–15, *p < 0.05 compared with respective MI/R) Abbreviations: WT, Wild type.

Having demonstrated that SF-PreCon-mediated cardioprotection is largely preserved in DM mice, we next determined whether such effects are mediated by AMPK, a pro-survival kinase impaired in diabetes. The effect of SF-PreCon upon MI/R injury was determined in cardiac-specific AMPKα2 dominant negative mice (AMPK-DN). Surprisingly, SF-PreCon preserved cardiac function in AMPK-DN mice (±Dp/dt: 21.7% and 22.2% compared to MI/R, P < 0.05 Fig. 3C; LVEF: decreased 15.9% compared to MI/R, P < 0.05, Fig. 3A;) and markedly increased in Longitudinal strain analysis (Fig. 3B), reduced infarct size (−15.9% vs MI/R, P < 0.05 Fig. 4A), and reduced apoptosis (−11.8% TUNEL stain positive cells compared to MI/R, P < 0.05, Fig. 4B; −27.7% caspase-3 activity vs MI/R, P < 0.05 Fig. 4C) in AMPK-DN mice. These results indicate that sevoflurane-mediated cardioprotection against MI/R is AMPK-independent.

### SF-PreCon differentially regulated MAPK family members in diabetic mice subjected to MI/R

**Figure 5:**
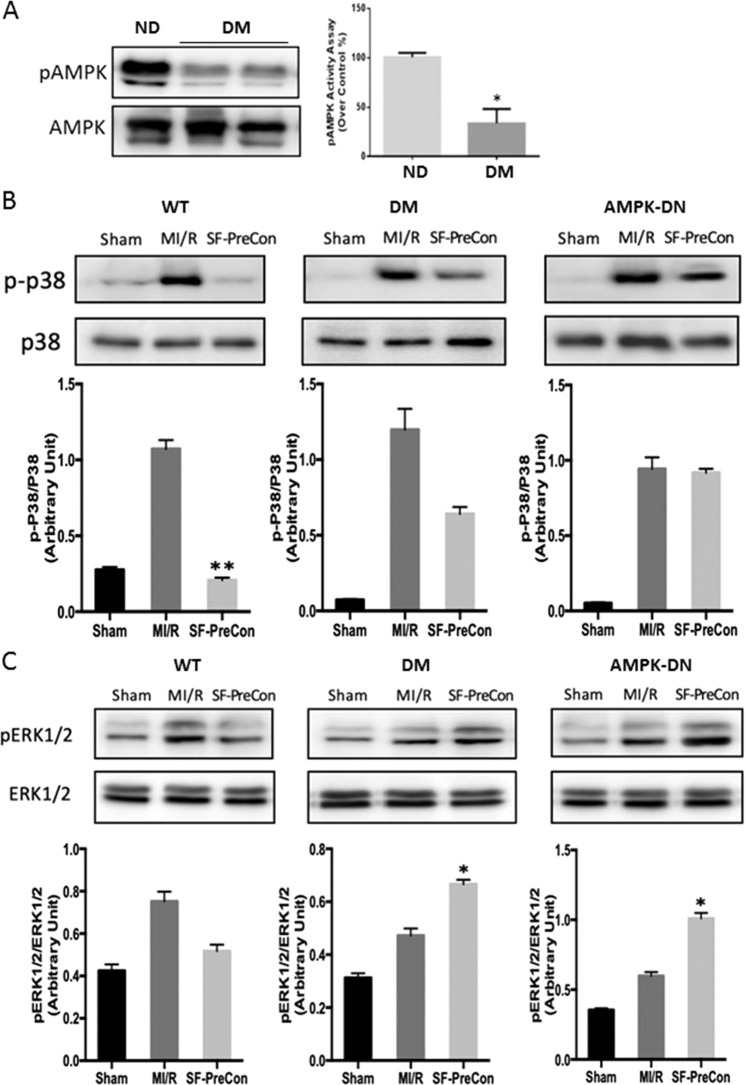
SF-PreCon ameliorates diabetic myocardial ischemia/reperfusion injury via differential regulation of p38 and ERK. (**A**) High-fat diet markedly reduced AMPK. (**B**) SF-PreCon reduced MI/R-induced activation of p38 in WT mice. The inhibitory effect of SF-PreCon upon p38 activation was blunted compared to WT, and virtually abolished in AMPK-DN mice. (**C**) SF-PreCon had no effect upon ERK1/2 phosphorylation in WT, but significantly increased phosphorylation of ERK1/2 in DM and AMPK-DN mice. (n = 10–12, *p < 0.05 compared to respective MI/R. **P < 0.01, compared with respective MI/R) Abbreviations: ND, Normal diet; DM, Diabetes.

As stated that AMPK signals was affected in diabetes (Fig. 5A) and to explore the molecular mechanisms responsible for SF-PreCon’s cardioprotective effect in DM mice, multiple molecules involved with cell survival were screened. Interestingly, SF-PreCon differentially regulated members of the mitogen-activated protein kinase (MAPK) family in the heart subjected to MI/R.

In ND mice, SF-PreCon markedly reduced (80.6% less than MI/R, P < 0.01) MI/R-induced activation of p38, a pro-death MAPK. Importantly, the inhibitory effect of SF-PreCon upon p38 activation was significantly blunted in DM mice. Furthermore, inhibition of p38 activation by SF-PreCon was virtually abolished in AMPK-DN mice (Fig. 5B).

**Figure 6:**
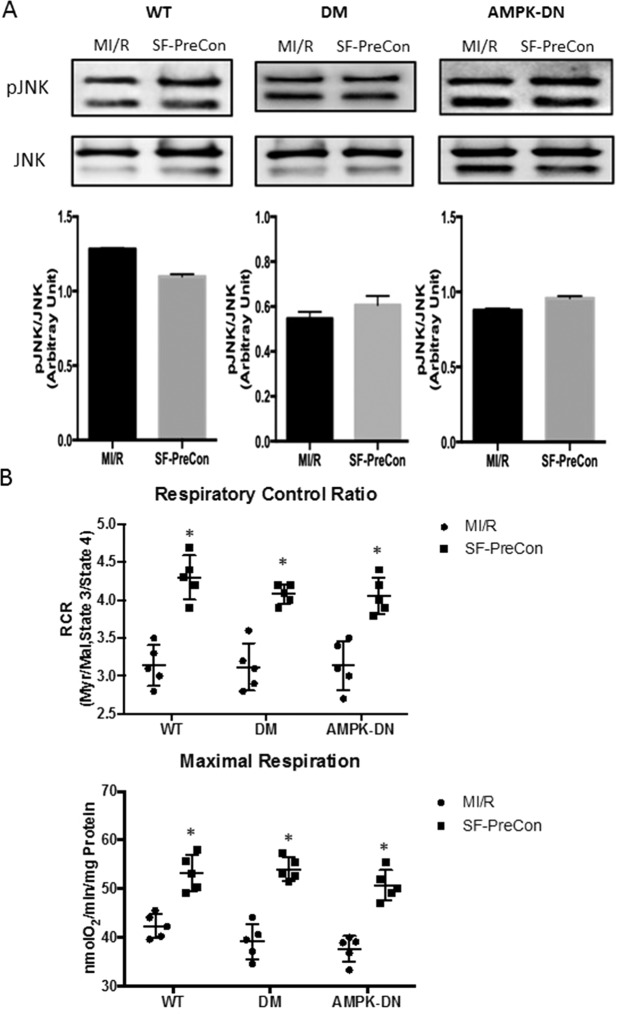
SF-PreCon augmented cardiac mitochondrial function in WT, DM, and AMPK-DN mice subjected to MI/R. (**A**) No significant SF-PreCon-induced effect upon JNK was observed in mice subjected to MI/R. (**B**) SF-PreCon significantly increased mitochondrial function in WT, DM, and AMPK-DN mice, evidenced by elevated respiratory control ratio and maximal respiration. (n = 8–12,*p < 0.05, compared to MI/R) Abbreviations: ND, Normal diet; DM, Diabetes.

Increased phosphorylation of the MAPK extracellular signal-regulated kinase 1/2 (ERK1/2, a pro-survival molecule) was observed in DM and AMPK-DN mice subjected to MI/R (41.1% and 68.8% respectively) treated by SF-PreCon. ERK1/2 was significantly downregulated in WT mice subjected to MI/R (Fig. 5C). No significant JNK activation was observed in mice treated with SF-PreCon subjected to MI/R (Fig. 6A).

Taken together, our results demonstrate SF-PreCon protects the heart via AMPK-dependent inhibition of pro-death MAPK (p38) in ND mice. However, SF-PreCon exerts its cardioprotective actions via AMPK-independent activation of the pro-survival MAPK (ERK1/2) in DM mice.

### SF-PreCon augmented cardiac mitochondrial function in WT, DM, and AMPK-DN mice subjected to MI/R

Apoptosis is a hallmark of MI/R injury. Mitochondria contribute largely to cardiomyocyte death in response to pathological stress induced by MI/R. We determined mitochondrial function in WT and diabetic mice subjected to MI/R. Compared to MI/R, SF-PreCon significantly increased mitochondrial function in WT and diabetic mice, evidenced by increased respiratory control ratio (1.37 and 1.31 fold increase compared to WT and diabetic mice respectively, P < 0.05, both after MI/R, Fig. 6B) and maximal respiration (25.9% and 37.8% increase compared to WT and diabetic mice respectively, P < 0.05, both after MI/R, Fig. 6B). AMPK dominant negative mice were treated with SF-PreCon and subjected to MI/R; SF-Precon again augmented mitochondrial function (1.29 fold increase compared to mice MI/R group, P < 0.05, Fig. 6B), giving further evidence the effect was independent of AMPK signaling.

## Discussion

**Figure 7:**
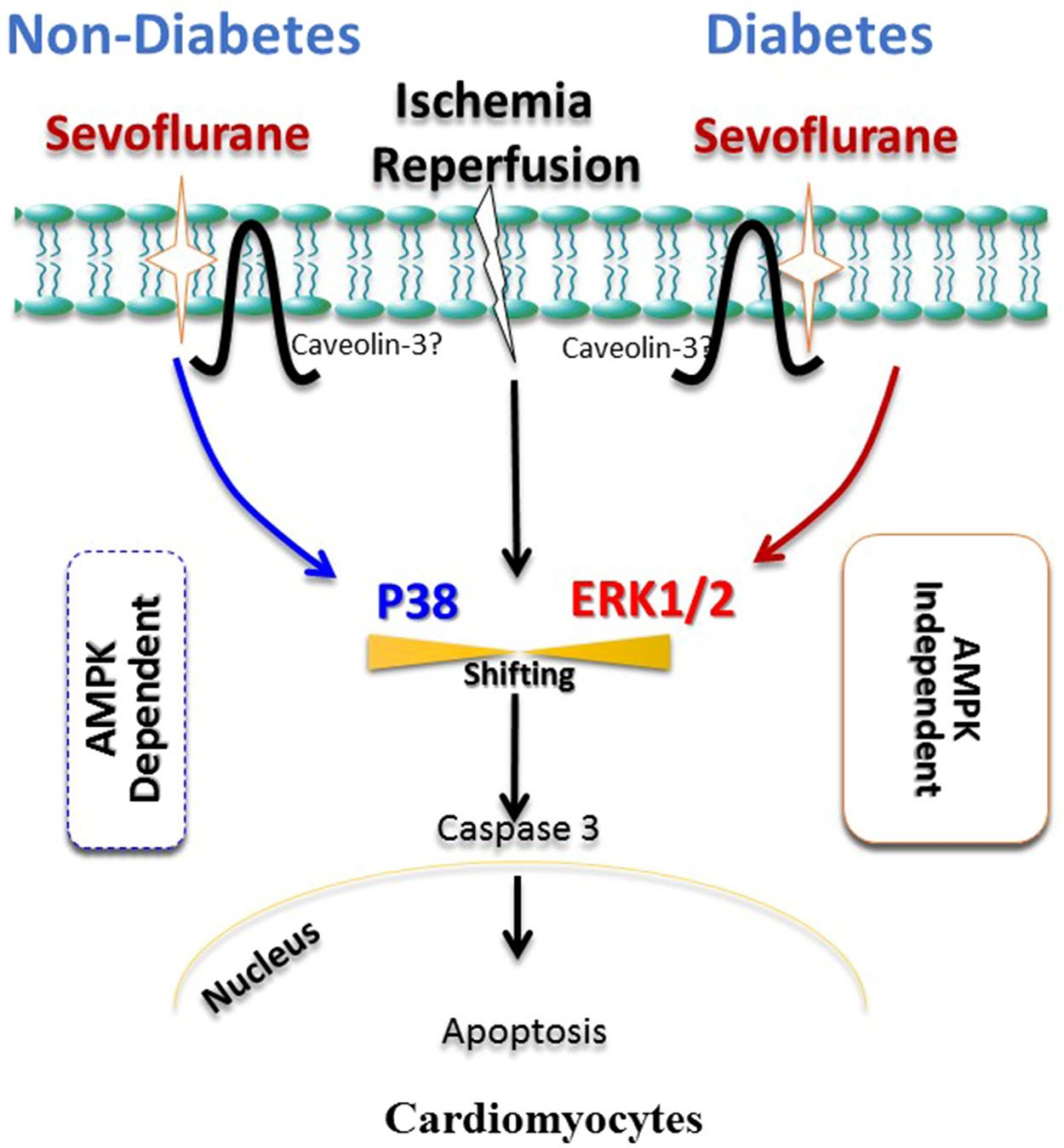
Diagram illustrating sevoflurane preconditioning diminishes MI/R injury via inhibition of pro-death MAPK pathway.

In the present study, we report sevoflurane preconditioning significantly ameliorates cardiac injury via activation of pro-survival MAPK in DM mice in an AMPK-independent manner. Yet, in ND mice, sevoflurane preconditioning diminishes MI/R injury via inhibition of pro death MAPK pathway (illustrated in Fig. 7). Additionally, SF-PreCon may protect the heart against MI/R injury during diabetes by augmenting mitochondrial function.

It is well accepted that SF-PreCon confers myocardial protection, resulting in markedly increased post-surgical cardiac index, reduced postoperative plasma cardiac troponin I (TnIc) levels, and decreased myocardial ischemia^10^. Whether volatile anesthetics have a cardioprotective effect in the diabetic condition is of great interest and clinical value. The clinical trials evaluating the cardioprotective effects of sevoflurane in diabetic patients with cardiac risk report agents such as sevoflurane are protective against myocardial ischemia in the perioperative setting. However, compelling evidence indicated no survival difference between diabetic and non-diabetic groups^11^. The observed response to preconditioning is not consistent. Therefore, studies targeted towards better understanding the mechanisms by which diabetes affects the beneficial effect of conditioning are needed. Different organ systems exhibit differential vulnerability and response to ischemia. In the heart, it is well recognized that cells subjected to I/R injury undergo cell death by apoptosis, a process strictly regulated by coordinated cellular signaling mechanisms. It may therefore be possible to salvage ischemic cells undergoing programmed, regulated cell death by interfering with involved apoptotic signaling pathways. Utilizing a high fat diet-induced diabetic mouse model, we demonstrated SF-PreCon markedly augmented cardiac function, decreased infarct size, and inhibited apoptosis.

The central role of AMPK in maintaining energy homeostasis has made it an attractive target in the investigation of metabolic diseases such as type 2 diabetes and obesity. In the current study, we demonstrated that a high-fat diet significantly decreased AMPK phosphorylation. We also employed a cardiomyocyte-specific APMKα2 dominant negative transgenic mouse model in our study experiments. Others and we have demonstrated that SF-PreCon activates AMPK, improves myocardial recovery, and ameliorates cardiac injury by a caveolin-3 modulated signaling pathway^12,13^. In consistent fashion, we confirmed the cardioprotective effect of SF-PreCon is largely preserved in AMPK-DN mice. Additionally, we demonstrated SF-PreCon cardioprotection remains intact in the diabetic condition, despite a compromised AMPK axis. The role of caveolin-3 was not addressed in the current study. Whether altered caveolin-3 interferes with the role of SF-PreCon in diabetes needs further investigation.

Given its branching communication with many other signaling networks, the MAPK family has garnered significant interest. Notably, MAPKs distinctly mediate cardiac development, metabolism, function, and pathology^14,15^. To address the signaling pathway responsible for SF-PreCon cardioprotection in the setting of diabetes, we detected MAPK family members in the heart subjected to MI/R. ERK, an important MAPK member, signaling provides cardioprotection against oxidative stress^16,17^. In our study, we demonstrated that SF-PreCon exerts cardioprotective effect by significantly increasing pERK1/2 levels in DM and AMPK-DN mice (underlining its AMPK-independent mechanism), while suppressing ERK1/2 activity in WT mice.

More importantly, the current study is the first to present evidence that SF-PreCon markedly reduced WT MI/R-induced activation of p38 (another MAPK, typically responding mostly to stressors such as oxidative, hyperosmotic, and radiation stress). The role of p38 is somewhat controversial in the literature. Whereas one study demonstrated p38 inhibition decreased cardiomyocyte apoptosis and improved cardiac function after MI/R^18^, others revealed p38 activation may confer cardioprotective effect^19,20^, mostly induced by ischemic preconditioning. Having demonstrated that SF-PreCon-mediated cardioprotection is significantly preserved in DM mice, we determined SF-PreCon did not inhibit p38 activation in DM or AMPK DN animals as in WT animals. SF-PreCon exhibited cardioprotection via differential regulation of MAPK family members, namely AMPK-dependent inhibition of pro-apoptotic MAPK (p38) and AMPK-independent increase of anti-apoptotic ERK activation.

In the current study, we also observed a trend of increased SF-PreCon-mediated activation of JNK in mice subjected to MI/R. Similar to p38, JNK plays a dual role in IR, mediating both protective and detrimental effects, dependent upon timing and severity of oxidative stress^21^. Such confounding results suggest that JNK may simultaneously and distinctly modulate both pro-and anti-apoptotic signaling pathways in heart^22–25^. Nevertheless, we report limited SP-PreCon induced upregulation of JNK activation in WT, DM, and AMPK-DN mice.

Having shown that SF-PreCon ameliorates diabetic MI/R injury by differential regulation of MAPK members (which directly interact with the outer mitochondrial membrane and translocate into mitochondria^26–28^, or indirectly affect mitochondria via ROS and calcium signaling^29–31^), we further demonstrated SF-PreCon significantly improves mitochondrial function in the WT and AMPK-DN heart subjected to MI/R. The precise mechanisms underlying the SF-PreCon-MAPK-mitochondria signaling pathway warrant further study, ongoing currently in our laboratory.

Previously, there has been much skepticism that sevoflurane preconditioning is favorable to cardiac function. Sevoflurane exhibited favorable effects in animal experiments, but clinical evidence indicated no survival difference between diabetic and non-diabetic groups^11^. However, Sevoflurane preconditioning cardioprotection is undoubted in clinical applications^32–34^. Our study demonstrates that preconditioning can directly positively influence cell survival signaling (MAPK) in the diabetic condition, rescuing the energetic pathway independently of endogenously suppressed metabolism. Additionally, sevoflurane has extra benefit in its support of the injured heart via mitochondrial apoptotic cascade regulation. Future studies investigating the clinical applicability of sevoflurane and providing more profound mechanisms in the diabetic population are warranted.

## Conclusion

In summary, we have demonstrated that SF-PreCon exerts cardioprotection, and inhibits p38 activation in an AMPK-dependent manner. In the setting of diabetes, SF-PreCon exerts cardioprotection and upregulates ERK1/2 activity in an AMPK-independent fashion. Although caution should be taken when extrapolating experimental findings to clinical practice, our report suggests that augmenting ERK1/2 activation may be an effective approach reducing perioperative cardiac injury in diabetic patients. Sevoflurane may represent an optimal anesthetic induction choice for patients with diabetes, a condition in which AMPK signaling is impaired.

## Methods

All protocols and experiments associated with this study were performed in strict adherence with the guidelines of the IACUC (Institutional Animal Care and Use Committee) at Tianjin Medical University, Shanxi Medical University, and Thomas Jefferson University.

### High-fat diet induced diabetes model

The high-fat diet induced type 2 diabetes model employed in this study was established as previously reported^35,36^. In brief, C57BL/6 J adult male mice (8–10 weeks, n = 10–15/group) were randomized to receive high-fat diet (HFD, 60%kcal, research Diets Inc. D12492i) or normal diet (ND, 10% kcal control, D12450Bi) containing the same protein content as HFD for 12 weeks.

### Animal and experiment setup

Cardiomyocyte specific AMPKα2 dominant negative expressing (AMPK-DN) and high-fat diet induced diabetic (DM, average body weight after HFD 41.11 ± 0.86 g) mice, along with each group’s respective wild-type (WT) littermates (4–5 months weeks old, average body weight 23.48 ± 0.51 g, n = 10–15/group), were utilized in this study. Prior to MI, animals were individually placed in an airtight Plexiglas anesthesia chamber. A calibrated vaporizer connected to the chamber delivered either 0% (control group) or 2% sevoflurane (SF-PreCon) gas mixture. Animals randomized to SF-PreCon treatment were exposed to 3 cycles of 10 minutes 2% sevoflurane periods interspersed with 15 minutes washout periods. Control animals were exposed to 3 cycles of 10 minutes 0% sevoflurane periods interspersed with 15 minutes washout periods. Subsequently, all mice were anesthetized with 2% isoflurane, and myocardial ischemia (MI) was induced by temporarily exteriorizing the heart and left anterior descending (LAD) coronary artery ligation via 6–0 silk suture slipknot as previously described^13^. Sham operated control mice (Sham) underwent the same surgical procedures, except the suture placed under the LAD was not tied. After 30 minutes of MI, the slipknot was released. Myocardial reperfusion (R) commenced for 3 hours (for signaling assay) or 24 hours (for cardiac function and infarct size measurement) as reported previously^13^. All assays utilized tissue from ischemic/reperfused regions or areas at risk (identified by Evans blue-negative staining).

### Determination of cardiac function, myocardial infarct size

Cardiac function was determined by hemodynamic assay (left ventricular catheterization via Millar 1.2 Fr micromanometer) and echocardiography (VisualSonic VeVo 2100, under 2% isoflurane anesthesia). Images were acquired in the short-axis B-mode and M-mode for analysis of cardiac function and dimensions. Long-axis B-mode images were recorded for longitudinal and radial strain analysis by VevoStrain software. Myocardial infarct size was determined by Evans blue-2,3,5-triphenyl tetrazolium chloride (1%, TTC) double staining. Briefly, the LAD was re-occluded and cannulated. Even’s blue dye was injected into the LAD and left atrium to delineate the anatomic area at risk (AAR, subjected to prolonged occlusion and reperfusion) and the non-ischemic normal zone. The heart was removed and sectioned in a serial transverse fashion. The unstained AAR was separated from the blue stained normal area. Slices were incubated at 37 °C for 20–30 minutes in 1% TTC in 0.1 mol/L phosphate buffer adjusted to pH 7.4, and photographed with a digital camera. Infarcted and non-infarcted myocardium within the AAR was digitally measured by image analysis software (Image J, version 1.47, National Institutes of Health, Bethesda, MD). Infarct size was expressed as a percentage of the AAR.

### Determination of myocardial apoptosis

Myocardial apoptosis was quantitatively analyzed by terminal deoxynucleotidyl transferase dUTP nick-end labeling (TUNEL) staining and caspase-3 activity assay as described previously^13^. The number of TUNEL-positive cardiomyocytes was counted in randomly selected high-power fields of the LV free wall at the mid-LV level from the endo- to epicardial portion. The percentage of TUNEL-positive cardiomyocytes was calculated by dividing the number of TUNEL-positive cardiomyocytes by the total number of cardiomyocytes observed in microscopic fields. Active caspase-3 was measured by assay kit on a SpectraMax-Plus microplate spectrophotometer (M5 Molecular Devices, Sunnyvale, CA).

### Western blot

Heart tissue was treated by lysis buffer (Cell Signaling) for protein extraction. Samples were loaded on 4–20% SDS-PAGE gels, transferred to PVDF membranes, and blotted in 5% film milk. PVDF membranes were probed with primary antibodies against AMPK, pAMPK, MAPK, and actin (Cell Signaling), and then incubated with secondary antibody for 1 hour. For protein detection, the Pierce ECL Substrate kit was used on a ChemiDoc MP Imager (Bo-Rad, CA). Western blots were quantified by densitometry (Image Lab).

### Mitochondrial function measurement

The cardiac mitochondrial function was analyzed using a Seahorse Bioscience XFe96 analyzer, as previously described^37^. Briefly, after cardiac function measurements (hemodynamic evaluation), heart tissue (400 mg) was harvested and homogenized in a prepared mitochondrial buffer (10 ml of 0.1 M Tris-MOPS and 1 ml of 0.1 M EGTA/Tris in 20 ml of 1 M sucrose). Then, the lysate was centrifuged for 10 minutes at 2000 RPM in 4 °C. The collected supernatant was centrifuged for 10 minutes at 5000 RPM in 4 °C. The mitochondrial buffer (50 μl) was added to the mitochondrial pellet sediment. After detecting the protein concentration with bio-ford method, 4 μg of mitochondrial protein was added to a collagen-coated plate. The plate was spun for 20 minutes at 2000 g on 4 °C, then sequentially loaded ADP, Oligomycin, FCCP, and Antimycin A in the Seahorse XFe96 FluxPak cartridge. The mitochondria coated plate and the cartridge were transferred to the XFe96 Extracellular Flux Analyzer (Seahorse Bioscience) for analysis.

### Statistical analysis

All results are presented as mean ± SD. Unless otherwise noted, data were evaluated by student’s t-tests for two groups or multiple groups with one-way ANOVA followed by Tukey’s multiple comparison post hoc test, using GraphPad Prism v7.0 software. P values less than 0.05 were considered statistically significant.

### Ethics approval and consent to participate

The IACUC Committee at Tianjin Medical University, Shanxi Medical University and Thomas Jefferson University approved the study.

## Data Availability

All data generated or analyzed during this study are included in this published article.
